# Supplementary material for: Differential modulation of the cellular and humoral immune responses in Drosophila is mediated by the endosomal ARF1-Asrij axis
Source: Sci Rep. 2017 Mar 8;7:118. doi: 10.1038/s41598-017-00118-7 (PMC5427928; doi:10.1038/s41598-017-00118-7)
Supplement: Supplementary file 1 — Inamdar Revised Supplemental Text [file 41598_2017_118_MOESM1_ESM.pdf]

Supplementary information:

Differential modulation of the cellular and humoral immune responses in *Drosophila* is mediated by the endosomal ARF1-Asrij axis.

Rohan J. Khadilkar†, Arindam Ray†, Chetan D.R., Arghyashree RoyChowdhury Sinha, Srivathsa S. Magadi, Vani Kulkarni and Maneesha S Inamdar*

Molecular Biology and Genetics Unit, Jawaharlal Nehru Centre for Advanced Scientific Research, Bangalore, India

*** Corresponding Author**

inamdar@jncasr.ac.in

† These authors contributed equally to this work.

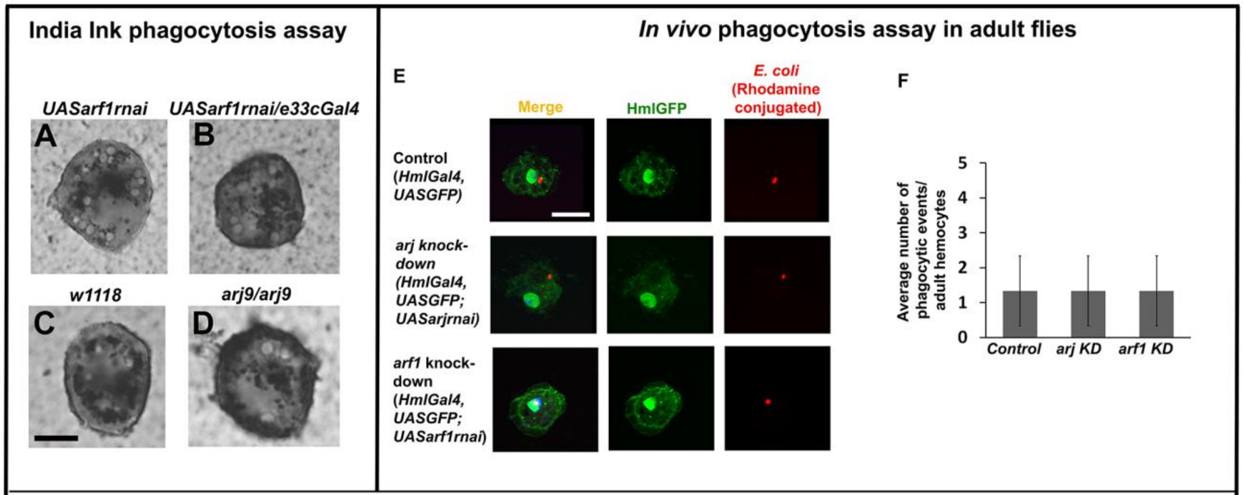

Supplementary Fig S1

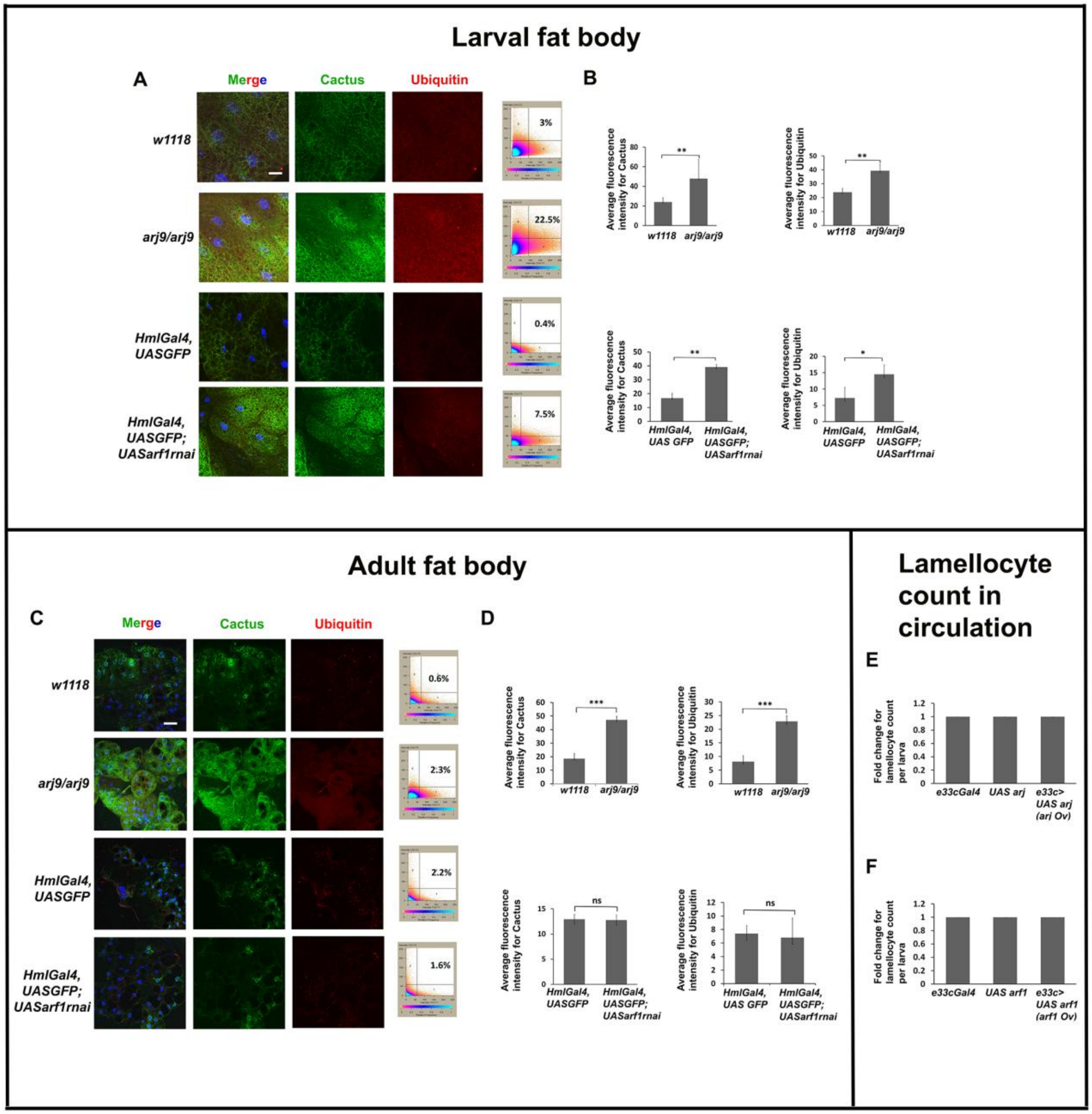

Supplementary Figure S2

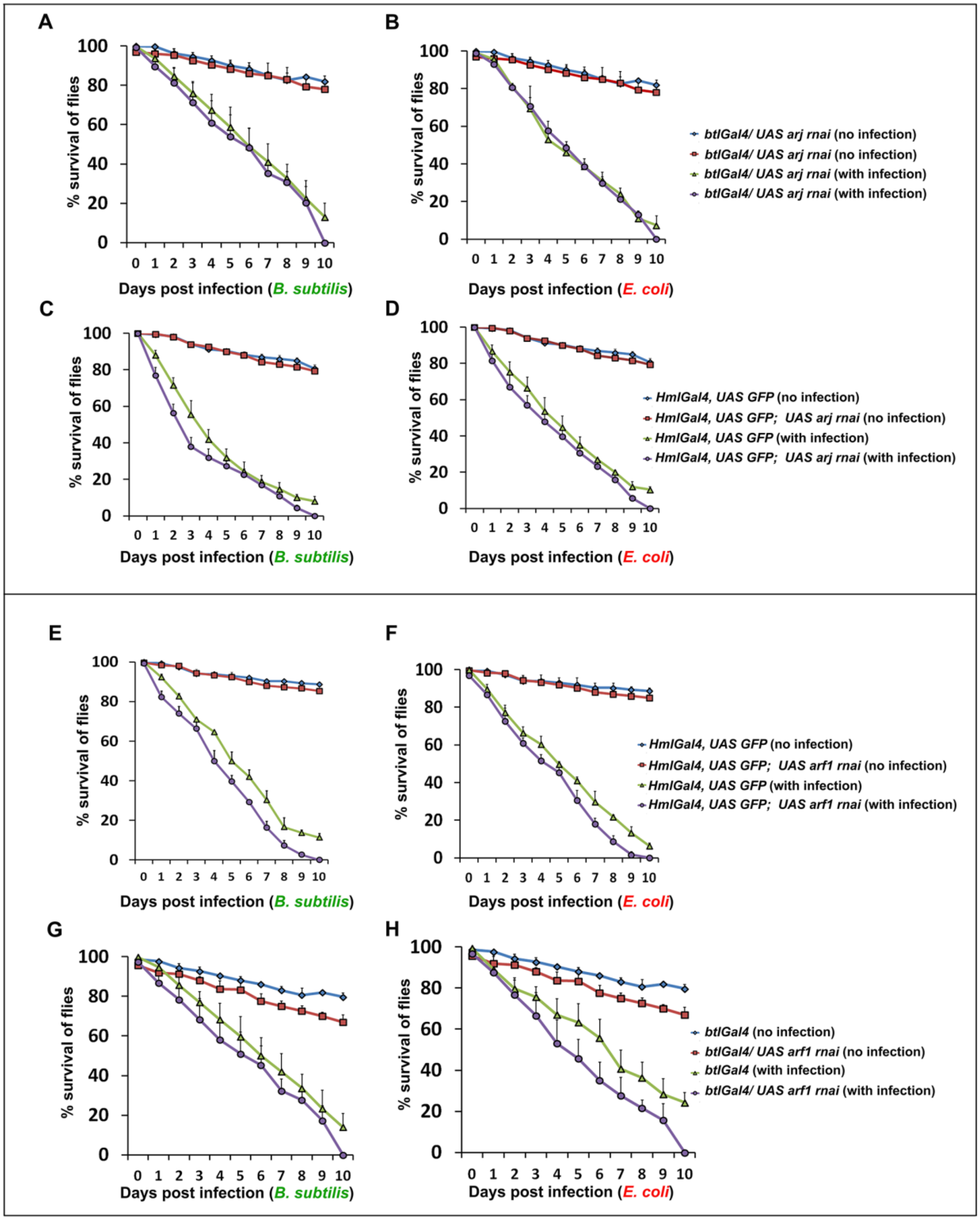

Supplementary Figure S3

Supplementary Figure S1: Asrij or ARF1 depletion does not cause any change in the phagocytic efficiency of the larval and adult hemocytes. (A-D) Images showing no change in India Ink uptake in *asrij* null (*arj9/arj9*) and ARF1 knockdown (*UAS arf1 rnai/e33CGal4*) larval hemocytes as compared to their respective controls *w1118* and *UAS arf1rnai*. (E) There is no change in the phagocytic uptake of Rhodamine conjugated heat killed *E. coli* particles in *asrij* knockdown (*HmlGal4,UASGFP; UASarjrnai*) or *arf1* knockdown (*HmlGal4, UASGFP; UAS arf1rnai*) adult hemocytes as compared to the control (*HmlGal4,UASGFP*). (F) Quantification of phagocytic events (number of bacteria internalised) per adult hemocytes. Scale Bar: (A-D) 5 µm (E) 10 µm. Error bars indicate standard error of mean.

Supplementary Figure S2: Asrij and ARF1 regulate Cactus ubiquitination in fat bodies and overexpression of either protein does not change lamellocyte count in larval circulation. (A) Images showing increased levels as well as higher co-localization percentages of Cactus and Ubiquitin in *asrij* null (*arj9/arj9*) or hemocyte specific *arf1* knockdown (*HmlGal4, UASGFP; UAS arf1rnai*) larval fat body as compared to the control, also indicated by adjacent co-localization plots. (B) Quantification of Cactus and Ubiquitin fluorescence intensity in fat bodies of control, *asrij* null and hemocyte specific *arf1* knockdown larvae (n=10). (C) Images showing increased levels of Cactus and ubiquitin in *asrij* null adult fat body as compared to the control, but no significant difference in *Hml>UAS arf1rnai* adult fat body as compared to its respective control. Adjacent co-localization plot shows increase in ubiquitination of Cactus in *asrij* null adult fat bodies but no change in case of fat bodies from hemocyte specific ARF1 knockdown flies. (D) Quantification of Cactus and Ubiquitin fluorescence intensity in fat bodies of control, *asrij* null and *HmlGal4* mediated *arf1* knockdown adult flies (n=10). Error bars indicate standard error of mean. * indicates P value<0.05, ** indicates P-value<0.01, *** indicates P-value<0.001 and ns indicates statistically non-significant difference. Scale bar: (A, C) 20 µm. (E) Quantification of fold change of lamellocyte count per larva upon Asrij overexpression (n=30). (F) Quantification of fold change of lamellocyte count per larva upon ARF1 overexpression (n=30).

Supplementary Figure S3: Hemocyte specific perturbation of Asrij or ARF1 affects the survival of flies upon bacterial infection. (A-H) Survival curves showing that *HmlGal4*-mediated *asrij* knockdown flies show reduced survival as compared to their respective controls upon infection with either *B. subtilis* (C) or *E.coli* (D). However trachea specific knockdown of *asrij* using *btlGal4* does not reduce survival (A, B). Reduction in survival ability is observed upon

*arf*1 knockdown in trachea in uninfected or infected condition(E, F) as well as upon *arf1* knockdown in hemocytes during infected condition (G, H). At least 100 flies were tested per genotype over at least three independent experiments.

Supplementary Table S1: PCR primer sets used for analysis

Primer Name	Sequence (5’ – 3’)	Location
attacin A F	CATCCTAATCGTGGCCCTG	Exon 1
attacin A R	CCATGACCAGCAT TGTTGTAG	Exon 1
cecropin A1 F	GTTTTCGTCGCTCTCATTCTG	Exon 1
cecropin A1 R	TTGTTGAGCGATTCCCAGTC	Exon 1
drosocin F	TCACCATCGTT TTCCTGCTG	Exon 1
drosocin R	TGATGGCAGCTTGAGTCAG	Exon 1
defensin F	TCT CGT GGC TAT CGC TTT TG	Exon 1
defensin R	CAG GCG GTG TGG TTC CAG	Exon 1
metchnikowin F	ATGCAACTTAATCTTGGAGCG	Exon 1
metchnikowin R	TAA ATT GGA CCC GGT CTT GG	Exon 1
drosomycin F	AGTACTTGTTCGCCCTCTTC	Exon 1
drosomycin R	TTAGCATCCTTCGCACCAG	Exon 1
diptericin F	ACCGCAGTACCCACTCAATC	Exon 1
diptericin R	CACTT TCC AGCTCG GTTCTG	Exon 1
rp49 F	CCGCTTCAAGGGACAGTATC	Exon 1-Exon 2 boundary
rp49 R	ACA ATC TCC TTG CGC TTC TTG	Exon 1-Exon 2 boundary
asrij F	TCCCTATCGCAACCATCGTG	Exon 1
asrij R	CGGGGAGTCCATGCTGATAC	Exon 1
